# Neutrophil-Lymphocyte and Platelet-Lymphocyte Ratios as Predictors of Complicated Acute Appendicitis: A Retrospective Cohort Study

**DOI:** 10.7759/cureus.105760

**Published:** 2026-03-24

**Authors:** Rafael Goncalves Nicastro, Bruno Amantini Messias, Yohran Cacciatori da Silva, Rodrigo Brasileiro de Souza, Julia Vitoria Nunes de Aquino, Priscila Benitz Rios de Oliveira, Erica Rossi Mocchetti, Cirenio de Almeida Barbosa, Marcelo Augusto Fontenelle Ribeiro Junior, Jaques Waisberg

**Affiliations:** 1 Department of Surgery, State Public Servant Hospital (IAMSPE), São Paulo, BRA; 2 Department of Surgery, General Hospital of Carapicuiba, Carapicuiba, BRA; 3 Medical School, São Camilo University Center, São Paulo, BRA; 4 Medical School, 9 de Julho University, São Paulo, BRA; 5 Department of Surgery, Ouro Preto University, Ouro Preto, BRA; 6 R Adams Cowley Shock Trauma Center, University of Maryland, Baltimore, USA; 7 Department of Surgery, ABC Medical School, Santo André, BRA

**Keywords:** acute appendicitis, biomarkers, neutrophil-lymphocyte ratio, platelet-lymphocyte ratio, risk stratification, severity

## Abstract

Background: Acute appendicitis (AA) is a frequent cause of acute abdominal pain in emergency departments. We aimed to evaluate the association and discriminatory performance of the neutrophil-lymphocyte ratio (NLR) and platelet-lymphocyte ratio (PLR) for identifying complicated acute appendicitis.

Methodology: In this retrospective study, we analyzed 483 patients treated at the General Hospital of Carapicuiba from January 2018 to June 2024. Normality was assessed using the Shapiro-Wilk test, and bivariate comparisons were performed using the Mann-Whitney test. Multivariate logistic regression was used to investigate the association of complicated AA with NLR, PLR, age, and sex. A receiver operating characteristic (ROC) curve was constructed to determine sensitivity, specificity, and the area under the curve (AUC). Statistical significance was set at *P* < 0.05.

Results: Of 483 patients, 273 (56.5%) had complicated appendicitis. Median NLR and PLR were higher in complicated versus uncomplicated cases (*P *< 0.001). The optimal ROC-derived cutoffs were NLR 8.84 (sensitivity 43.6%, specificity 79.5%; AUC 0.694; 95% confidence interval (CI), 0.645-0.742) and PLR 170.6 (sensitivity 40.7%, specificity 73.3%; AUC 0.600; 95% CI, 0.550-0.651). In unadjusted analysis, NLR >8.84 was associated with complicated appendicitis (odds ratio (OR) 3.00; 95% CI, 1.99-4.53). In the multivariable model including age and sex, PLR remained independently associated with complicated appendicitis.

Conclusions: NLR and PLR were associated with complicated appendicitis in unadjusted analyses. After adjustment for age and sex, only PLR remained independently associated. Given their low cost and wide availability, these ratios may complement clinical assessment to support risk stratification and timely management, particularly in resource-limited settings.

## Introduction

Acute appendicitis (AA) is a common inflammatory condition of the vermiform appendix, with an estimated prevalence of 7%-8% in the general population and comprising approximately 60% of all acute abdominal cases presenting to the emergency department [1-8].

AA is classified as uncomplicated or complicated, with most cases being uncomplicated (70%-80%). Complications, including appendiceal rupture, occur in approximately 20% of patients [1-3]. Such complications are associated with necrotic and ruptured appendices and may progress to purulent or fecal peritonitis [1]. Timely diagnosis is crucial, as delays can increase the risk of progression to complicated AA, thereby elevating morbidity and mortality rates [1-3,9-12]. Consequently, accurate assessment of severity is vital to prevent fatal outcomes [4].

Diagnosis is primarily based on clinical history and physical examination [1,3]. It is supported by scoring systems such as the Alvarado, Raja Isteri Pengiran Anak Saleha, right iliac fossa pain, and Appendicitis Inflammatory Response scores [1,5,6]. However, these scoring systems have limitations, primarily owing to their lack of specificity and inability to effectively stratify patients into uncomplicated and complicated categories [1,6,8].

In recent years, imaging examinations have played an increasingly prominent role in enhancing the accuracy of appendicitis diagnosis [9]. Among these modalities, computed tomography (CT) scans have the highest diagnostic accuracy. CT scans provide adequate differentiation between non-ruptured and ruptured AA; however, they are not widely available in low- and middle-income hospitals, thereby limiting effective stratification in some cases [8]. Therefore, improving the diagnostic accuracy of biomarkers could help healthcare professionals diagnose complicated AA and reduce the risk of postoperative complications [9].

Several serum markers have been examined as predictors of AA severity. Common markers include leukocyte count, bilirubin, C-reactive protein (CRP), and sodium levels [1]. Most patients with AA present with leukocytosis, which alone is insufficient to distinguish between uncomplicated and complicated cases [1,6,8,10,13]. Elevated serum bilirubin levels have been identified as a potential marker for ruptured appendices; however, they exhibit low sensitivity and specificity [1,8]. CRP has shown better performance than bilirubin in predicting rupture, but is not considered an effective standalone test [1,8,10,13]. Serum sodium level is another marker being investigated, with hyponatremia effectively predicting the severity of AA [13,14]. Recent studies have reported that hematologic parameters, such as the neutrophil-lymphocyte ratio (NLR) and platelet-lymphocyte ratio (PLR), are effective inflammatory markers for the diagnosis and assessment of AA severity [1,4,6,10,12]. NLR and PLR are simple, cost-effective, and easily interpretable using widely available tests, such as the complete blood count (CBC) [15].

The NLR reflects two immunologic phases: the innate response, signified by neutrophils, and the adaptive response, characterized by lymphocytes [15]. Neutrophils, the primary effector cells of the systemic inflammatory response (SIR), rapidly increase in number following the onset of inflammation. This increase in neutrophils and the concurrent decrease in circulating lymphocytes result from the release of endogenous cortisol and catecholamines owing to immune system activation during inflammatory stress [1,15,16]. Lymphopenia subsequently occurs owing to lymphocyte migration to the reticuloendothelial system [12,16]. Additionally, the inflammatory cascade triggers the release of proinflammatory cytokines, which stimulate megakaryocyte proliferation, thereby increasing the number of circulating platelets. This increase in platelets elevates the PLR, in contrast to the decrease in lymphocytes [7].

The NLR may increase under conditions that cause tissue damage and activate the SIR. An increase in the NLR within <6 hours identifies this ratio as a marker of acute stress, detectable before other markers such as CRP [15]. In addition to its mediating role in the SIR, the NLR is a prognostic factor for morbidity and mortality in various diseases; however, well-established reference ranges are lacking [15].

Several studies have aimed to identify markers of AA severity [1,10,14]; however, no biomarker has yet been identified that can be used for effective diagnosis or prediction of AA severity [5,6]. Considering these gaps and the need for simple tools in settings with limited access to imaging, the objectives of this study were to evaluate the association and discriminatory performance of NLR and PLR for identifying complicated acute appendicitis (CAA) and to explore whether these biomarkers could support clinical risk stratification as adjunctive tools.

## Materials and methods

A retrospective cohort study was conducted in the Surgery Department of the General Hospital of Carapicuiba to analyze whether the NLR and PLR could be used to distinguish between complicated and uncomplicated AA. This study included 483 patients who had undergone an appendectomy between January 2018 and June 2024. The Research Ethics Committee of São Camilo University Center approved the study (CAAE: 75345823.1.0000.0062), and the requirement for informed consent was waived owing to the study’s retrospective design. All patients diagnosed with acute appendicitis were managed surgically according to the institutional protocol during the study period. Per local guidelines, the management of choice was appendectomy; therefore, no patient was treated non-operatively.

Demographic and clinical data (age, sex, intraoperative findings, histopathological results, and neutrophil, lymphocyte, and platelet counts from the initial laboratory evaluation performed at admission) were collected from the patients’ electronic medical records. Although these hematologic parameters were obtained from the admission workup and used for preoperative assessment, the exact time of blood collection was not consistently available in the medical records. Patients of both genders and all suspected cases of AA were included, whereas patients who were immunosuppressed, pregnant, without histopathological confirmation of appendicitis, or had surgeries associated with appendectomy were excluded. Only patients with complete data for the variables included in the analysis were evaluated; no data imputation was performed. Age and sex were included in the multivariable model as clinically relevant covariates defined a priori, whereas NLR and PLR were the biomarkers of primary interest.

The NLR and PLR were calculated by dividing the number of neutrophils and platelets by the number of lymphocytes, respectively. These ratios were analyzed in blood tests requested preoperatively. NLR and PLR were initially analyzed as continuous variables and subsequently dichotomized using optimal ROC-derived cutoffs for secondary analyses.

Patients were categorized into two groups: complicated appendicitis (*n* = 273) and uncomplicated appendicitis (*n* = 210). Complicated appendicitis was defined as the presence of appendiceal necrosis or perforation, purulent peritonitis, or abscess intraoperatively. Patients without these complications were categorized as having uncomplicated AA.

Statistical analyses were performed using SPSS version 26 (IBM Corp., Armonk, NY) software. The Shapiro-Wilk test was used to assess data normality. Continuous variables were summarized as medians and interquartile ranges and compared between complicated and uncomplicated appendicitis using the Mann-Whitney U test. Categorical variables were presented as frequencies and percentages and compared using Pearson’s chi-square test. Univariable logistic regression analyses were performed for dichotomized NLR and PLR using receiver operating characteristic (ROC)-derived cutoffs, and multivariable logistic regression analyses (Enter and exploratory stepwise methods) were used to explore the associations between complicated AA and NLR, PLR, age, and sex. Wald statistics were used to test coefficients in the logistic regression models. ROC curves were constructed, and sensitivity, specificity, positive predictive value (PPV), negative predictive value (NPV), area under the curve (AUC), and 95% confidence intervals (CIs) were calculated. The significance level was set at *P* < 0.05. This study was reported in accordance with the STROBE statement.

## Results

During the study period, 483 patients were included; 273 (56.5%) had complicated appendicitis and 210 (43.5%) had uncomplicated appendicitis. Most patients were male (295, 61.1%), and the proportion of complicated appendicitis did not differ by sex (*P* = 0.539) (Table 1).  

**Table 1:** Association between AA and sex. The *P*-value corresponds to the comparison of sex distribution between complicated and uncomplicated appendicitis (*P* = 0.539). Pearson’s chi-square test was used (χ² = 0.377). AA, acute appendicitis; *n*, number

	Complicated		Uncomplicated		Total	
	*n*	%	n	%	n	%
Female	103	37.7%	85	40.5%	188	38.9%
Male	170	62.3%	125	59.5%	295	61.1%
Total	273	56.5%	210	43.5%	483	100.0%

The median patient age was 21 years, and no significant differences were observed between age and appendicitis type (*P* = 0.448). However, the median NLR was significantly higher in patients with complicated AA than in those with uncomplicated AA (complicated AA, 7.73; uncomplicated AA, 4.52; *P* < 0.001). Similarly, there was a significant difference in PLR between the complicated and uncomplicated groups (complicated AA, 151.9; uncomplicated AA, 122.1; *P* < 0.001) (Table 2).

**Table 2:** Association of age, NLR, and PLR with AA. The Mann-Whitney U test was used for all comparisons; the standardized *Z* statistic is shown. AA, acute appendicitis; IQR, interquartile range; N, number; NLR, neutrophil-lymphocyte ratio; PLR, platelet-lymphocyte ratio; Q1, first quartile; Q3, third quartile

		Median	Q1	Q3	IQR	*n*	*P*-value	*Z *statistic
Age	Complicated	21	13	36	23	273	0.448	0.759
	Uncomplicated	21	13	29	16	210		
NLR	Complicated	7.73	5.27	12.00	6.73	273	<0.001	8.220
	Uncomplicated	4.52	3.00	7.83	4.83	210		
PLR	Complicated	151.9	104.7	225.8	121.1	273	<0.001	3.900
	Uncomplicated	122.1	94.4	181.2	86.9	210		

**Figure 1:**
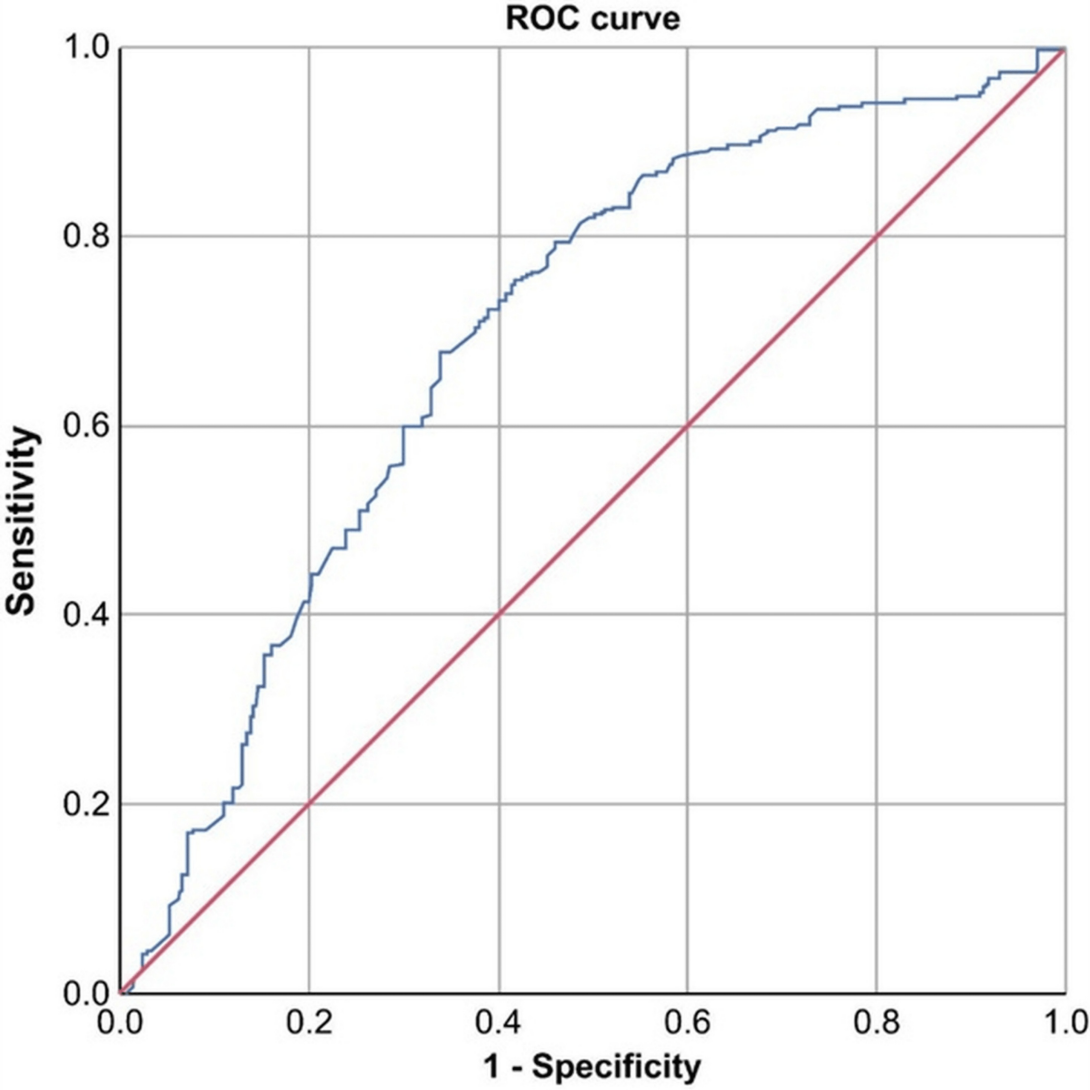
ROC curve of NLR for predicting complicated appendicitis. The AUC was 0.694 (95% CI, 0.645-0.742; *P* < 0.001). NLR, neutrophil-lymphocyte ratio; ROC, receiver operating characteristic; AUC, area under the curve

**Figure 2:**
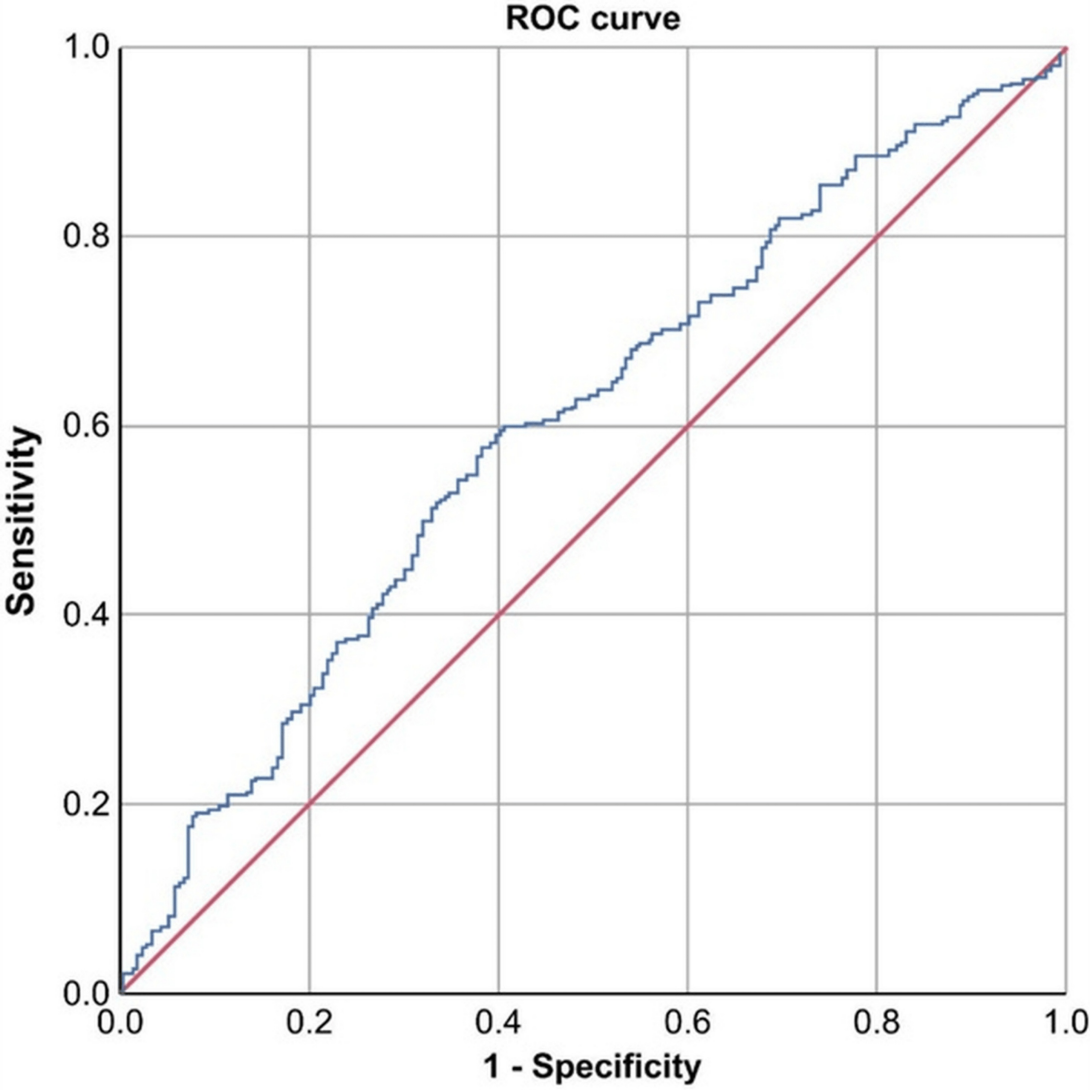
ROC curve of PLR for predicting complicated appendicitis. The AUC was 0.600 (95% CI, 0.550-0.651; *P* < 0.001). PLR, platelet-lymphocyte ratio; ROC, receiver operating characteristic; AUC, area under the curve

ROC analysis yielded an optimal cutoff of 8.84 for NLR (sensitivity, 43.6%; specificity, 79.5%; PPV, 73.5%; NPV, 52%; AUC, 0.694; 95% CI, 0.645-0.742; *P* < 0.001) and 170.6 for PLR (sensitivity, 40.7%; specificity, 73.3%; PPV, 66.5%; NPV, 48.7%; AUC, 0.600; 95% CI, 0.550-0.651; *P* < 0.001) (Table 3, Figures 1-2).

**Table 3:** Diagnostic performance of ROC-derived NLR and PLR cutoffs for complicated AA. The table summarizes the diagnostic performance of the optimal ROC-derived cutoffs (NLR = 8.84; PLR = 170.6). AA, acute appendicitis; AUC, area under the curve; CI, confidence interval; NLR, neutrophil-lymphocyte ratio; NPV, negative predictive value; PLR, platelet-lymphocyte ratio; PPV, positive predictive value

Metric	NLR	PLR
Cutoff	8.84	170.6
AUC	0.694	0.600
95% CI	0.645-0.742	0.550-0.651
Sensitivity	43.6%	40.7%
Specificity	79.5%	73.3%
PPV	73.5%	66.5%
NPV	52%	48.7%

In the multivariable ENTER model including male sex, age, NLR, and PLR, only PLR remained independently associated with complicated appendicitis (OR 1.002; 95% CI, 1.000-1.005; *P* = 0.015), whereas NLR was not significant (OR 1.001; 95% CI, 0.982-1.021; *P* = 0.909). Age showed a tendency toward association (OR 1.012; 95% CI, 1.000-1.024; *P* = 0.054), and male sex was not independently associated with the outcome. In an exploratory stepwise model, which retained the most informative variables, PLR remained an independent predictor (OR 1.003; 95% CI, 1.001-1.004; *P* = 0.003), while age showed only a trend (*P* = 0.065). Overall, PLR emerged as the only independent predictor of complicated AA in multivariable analysis, whereas NLR showed a strong univariable association but lost significance after adjustment. Details of the multivariable analysis are shown in Table 4. In unadjusted analysis, NLR > 8.84 was associated with complicated appendicitis (OR 3.00; 95% CI, 1.99-4.53; *P* < 0.001).

**Table 4:** Multivariate logistic regression model for complicated AA. Enter = forced-entry multivariable logistic regression including all prespecified covariates (male sex, age, NLR, and PLR). Stepwise = exploratory stepwise multivariable logistic regression retaining variables according to the software selection procedure. Lower and upper limits represent the 95% CI for the OR. Hyphens indicate variables not retained in the stepwise model. AA, acute appendicitis; Coef., coefficient; CI, confidence interval; NLR, neutrophil-lymphocyte ratio; OR, odds ratio; PLR, platelet-lymphocyte ratio

	Enter						Stepwise					
	Coef. (B)	Wald	*P*-value	OR			Coef. (B)	Wald	*P*-value	OR		
				OR	Lower limit	Upper limit				OR	Lower limit	Upper limit
Male	0.172	0.794	0.373	1.187	0.814	1.733	-	-	-	-	-	-
Age	0.012	3.714	0.054	1.012	1.000	1.024	0.011	3.413	0.065	1.011	0.999	1.023
NLR	0.001	0.013	0.909	1.001	0.982	1.021	-	-	-	-	-	-
PLR	0.002	5.901	0.015	1.002	1.000	1.005	0.003	8.667	0.003	1.003	1.001	1.004

## Discussion

AA is among the most prevalent causes of acute abdominal pain in emergency departments [17-19]. It is primarily diagnosed clinically; however, imaging examinations, especially CT, have increasingly been used to assess abdominal pain owing to their high diagnostic accuracy in identifying AA-related complications [20-23]. Owing to the limited availability of these imaging services in emergency settings and the risks associated with ionizing radiation, alternative biomarkers, such as the leukocyte count, CRP, bilirubin and sodium levels, the NLR, and the PLR, are also used to differentiate between complicated and uncomplicated AA [19,22,24].

Timely identification of complicated AA significantly influences decision-making and the appropriate allocation of resources for patients with complicated disease [24]. Prompt surgical intervention is crucial in complicated cases because delays can lead to rupture rates of up to 40% [24-29].

The NLR and PLR indicate two distinct immunological phases: innate and adaptive responses [7,15]. During acute inflammation, the innate immune response escalates neutrophil levels by releasing various proinflammatory substances that regulate immunity [15]. Conversely, the adaptive response involves lymphocyte marginalization in the reticuloendothelial system, thereby reducing serum levels [16]. The platelet count increases as a result of recruitment mediated by proinflammatory cytokines released in response to acute stress [7]. Elevated NLRs and PLRs correlate with acute inflammatory diseases and are directly proportional to disease severity, as observed in complicated AA cases [30-35].

Several studies have examined the NLR and PLR as predictors of complicated AA. In a retrospective study involving 799 patients aged ≥16 years undergoing appendectomy, Rajalingam et al. identified an NLR threshold >6.96 as a predictor of complicated AA, with an AUC of 0.727, 26.5% sensitivity, 91.6% specificity, 69.3% PPV, and 63.7% NPV [1]. Hajibandeh et al. conducted a systematic review and meta-analysis involving 8,914 patients and reported an NLR >8.8, with 76.92% sensitivity, 100% specificity, and an AUC of 0.91 for predicting complicated AA [29]. Our study identified a distinct cutoff point of 8.84 for the NLR (sensitivity and specificity: 43.6% and 79.5%, respectively), with an AUC of 0.694 (*P* < 0.001), a PPV of 73.5%, and an NPV of 52%. An increased NLR is known to be associated with an elevated risk of complicated AA [1,5,12,19,29,31,35-37] and is correlated with the histopathological grade of appendicitis [11,38].

Esquivel-Esquivel et al. used an NLR >7.41 (OR 3.82, 95% CI, 1.88-7.79) as a cutoff value, and Mori et al. used a value >11.4 (OR 3.65, 95% CI, 1.230-10.863) to prognosticate complicated AA [2,12]. These studies presented OR values similar to those identified in our study (OR 3.00, 95% CI, 1.99-4.53, *P* < 0.001), indicating that patients with a high NLR are three times more likely to progress to complicated AA. However, studies by Yesilalioglu et al. and Vargas Rodríguez et al. did not establish a correlation between an increased NLR and a higher risk of complicated AA (OR 1.22, 95% CI, 0.82-1.52) [5,10].

Although NLR has been the most extensively evaluated ratio in the literature, our findings underscore the relevance of PLR as a potentially more robust marker in adjusted analyses. In our study, PLR emerged as the only independent predictor after adjustment for age and sex. Some studies have reported the effectiveness of the PLR as a marker of AA severity. Rajalingam et al. reported that a PLR with a cutoff value of 180.5 had 22.4% sensitivity, 89.0% specificity, a PPV of 60.6%, an NPV of 60.4%, and an AUC of 0.653 for predicting complicated AA (OR 2.351, 95% CI, 1.594-3.465) [1]. In a study by Cruz-Vallejo et al., the PLR had 77.7% sensitivity, 63.5% specificity, and an OR of 2.87 (95% CI, 1.54-5.34), with a cutoff value >284 [34]. In our study, the PLR showed 40.7% sensitivity, 73.3% specificity, and an OR of 1.88 (95% CI, 1.28-2.78, *P* < 0.001) with a cutoff value of 170.6, aligning with the findings of Rajalingam et al. [1] and Cruz-Vallejo et al. [34].

Another study reported a higher PLR in cases of ruptured appendices, making it a more sensitive marker of inflammation than the serum leukocyte count and the NLR [11]. However, Mekrugsakit et al. reported that the NLR had no statistical significance in differentiating complicated from uncomplicated AA [36]. Ayeni et al. proposed that, when combined, the PLR and NLR are synergistic markers that significantly enhance the differentiation between complicated and uncomplicated AA [35].

The NLR and PLR could help in prioritizing surgical indications in patients with AA when CT is unavailable [1,24]. The use of the NLR and PLR offers several benefits in surgical practice owing to their low cost, ease of calculation within the CBC, wide availability, and simplicity of interpretation, in addition to increasing earlier than markers such as CRP [1,9,12,15]. Therefore, these ratios could serve as adjunct markers to help healthcare professionals in low- and middle-income countries, or those without access to CT, in diagnosing complicated AA and in resource allocation and decision making [1,39].

In summary, despite a lack of consensus concerning cutoff values for the NLR and PLR because of methodological disparities across study populations, variability in CBC results, and sample demographics, both ratios show promise as predictors of complicated AA, with higher values indicating more severe disease [1,2,11,12,34,35]. From a pragmatic perspective, our findings suggest that the NLR and PLR may be used as adjunct markers, but their modest discriminatory performance indicates that they should not be used in isolation for clinical decision-making. Rather, incorporation of these ratios into broader clinical and laboratory assessment, and potentially into composite scoring systems, may offer greater value than standalone use.

The limitations of this study include its retrospective design, single-center sample, the use of different cutoff values for the analyzed ratios, and the consecutive surgical cohort, which delimits the scope of inference to patients treated surgically. Because the cohort included only surgically treated patients, caution is warranted when extending these results to non-operative settings. Although complicated appendicitis was classified using predefined intraoperative criteria, some variability in surgical interpretation may have occurred. In addition, the lack of external validation suggests that the proposed cutoff values may be cohort-specific. Variations in symptom duration before presentation, together with the modest discriminatory performance observed and the potential collinearity between NLR and PLR, further support the use of these biomarkers as adjunctive rather than standalone tools. Therefore, more randomized multicenter studies concerning complicated and uncomplicated AA are needed to confirm our findings and effectively translate them into clinical practice.

## Conclusions

NLR and PLR were associated with complicated acute appendicitis in unadjusted analyses, while only the PLR remained independently associated after adjustment for age and sex. Given their low cost and wide availability, these ratios may complement clinical assessment, but their modest discriminatory performance indicates that they should be interpreted as adjunctive rather than standalone tools for risk stratification.
